# Factors Associated With Telemedicine Use Among German General Practitioners and Rheumatologists: Secondary Analysis of Data From a Nationwide Survey

**DOI:** 10.2196/40304

**Published:** 2022-11-30

**Authors:** Felix Muehlensiepen, Pascal Petit, Johannes Knitza, Martin Welcker, Nicolas Vuillerme

**Affiliations:** 1 Center for Health Services Research Faculty of Health Sciences Brandenburg Brandenburg Medical School Theodor Fontane Rüdersdorf bei Berlin Germany; 2 AGEIS Université Grenoble Alpes Grenoble France; 3 Department of Internal Medicine 3 Friedrich-Alexander-University Erlangen-Nürnberg and Universitätsklinikum Erlangen Erlangen Germany; 4 Medizinisches Versorgungszentrum für Rheumatologie Dr M Welcker GmbH Planegg Germany; 5 Institut Universitaire de France Paris France; 6 LabCom Telecom4Health Orange Labs & Université Grenoble Alpes CNRS, Inria Grenoble France

**Keywords:** telemedicine, rheumatology, primary care, secondary analysis, health services research

## Abstract

**Background:**

Previous studies have demonstrated telemedicine (TM) to be an effective tool to complement rheumatology care and address workforce shortage. With the outbreak of the SARS-CoV-2 pandemic, TM experienced a massive upswing. However, in rheumatology care, the use of TM stagnated again shortly thereafter. Consequently, the factors associated with physicians’ willingness to use TM (TM willingness) and actual use of TM (TM use) need to be thoroughly investigated.

**Objective:**

This study aimed to identify the factors that determine TM use and TM willingness among German general practitioners and rheumatologists.

**Methods:**

We conducted a secondary analysis of data from a German nationwide cross-sectional survey with general practitioners and rheumatologists. Bayesian univariate and multivariate logistic regression analyses were applied to the data to determine which factors were associated with TM use and TM willingness. The predictor variables (covariates) that were studied individually included sociodemographic factors (eg, age and sex), work characteristics (eg, practice location and medical specialty), and self-assessed knowledge of TM. All the variables positively and negatively associated with TM use and TM willingness in the univariate analysis were then considered for Bayesian model averaging analysis after a selection based on the variance inflation factor (≤2.5). All analyses were stratified by sex.

**Results:**

Univariate analysis revealed that out of 83 variables, 36 (43%) and 34 (41%) variables were positively or negatively associated (region of practical equivalence≤5%) with TM use and TM willingness, respectively. The Bayesian model averaging analysis allowed us to identify 13 and 17 factors of TM use and TM willingness, respectively. Among these factors, being female, having very poor knowledge of TM, treating <500 patients per quarter, and not being willing to use TM were negatively associated with TM use, whereas having good knowledge of TM and treating >1000 patients per quarter were positively associated with TM use. In addition, being aged 51 to 60 years, thinking that TM is not important for current and future work, and not currently using TM were negatively associated with TM willingness, whereas owning a smart device and working in an urban area were positively associated with TM willingness.

**Conclusions:**

The results point to the close connection between health care professionals’ knowledge of TM and actual TM use. These results lend support to the integration of digital competencies into medical education as well as hands-on training for health care professionals. Incentive programs for physicians aged >50 years and practicing in rural areas could further encourage TM willingness.

## Introduction

Telemedicine (TM) offers the opportunity to overcome spatial distances in health care delivery [1]. Thus, TM represents a promising way to support rheumatology care [2,3] in light of the rising worldwide burden of musculoskeletal diseases [4] and growing workforce shortage [5,6]. However, the effective implementation of TM in standard care is only possible if the end users are willing and able to use TM [7,8].

With the outbreak of the SARS-CoV-2 pandemic, physicians’ face-to-face consultations declined considerably [9,10]. The possibility of contactless medical care is now more important. Advantageously, through TM, medical care could be provided, avoiding contacts and thus infections [11,12]. Hence, TM has received a tremendous upswing worldwide [13] and regionally [9,14]. Although the pandemic situation, involving social distancing and multiple lockdowns, provided an ideal environment for the implementation of TM, this momentum soon stagnated again [10,15]. Particularly in rheumatology, health care professionals’ use and acceptance of TM fell short of expectations [10]. Apparently, other factors may play a role in the willingness to use TM (TM willingness) and actual use of TM (TM use) among general practitioners (GPs) and rheumatologists. Identifying these factors is a rather challenging task but could have implications for the development of TM strategies aiming to improve health outcomes and access to care and make health care delivery systems more efficient and cost-effective.

To gain a better understanding of these factors, we performed a secondary analysis using data from a nationwide cross-sectional survey conducted earlier in Germany [7]. Our objective was to identify the underlying factors associated with TM use and the TM willingness among German GPs and rheumatologists.

## Methods

### Overview

This work reports on findings from a secondary analysis of data collected as part of a cross-sectional, self-completed, and paper-based survey of German GPs and outpatient rheumatologists. The initial study was conducted from September to November 2018 and investigated the acceptance, opportunities, and obstacles to the implementation of TM. Of the 2395 questionnaires that were sent out, 497 (20.75%) were returned. Of the 497 responses, 12 (2.4%) were excluded from the data set because fewer than half of the questions were answered. The final response rates were 18.94% (437/2307) and 55% (48/88) for GPs and rheumatologists, respectively. The exact methodology applied for the nationwide survey has been described previously [7].

### Regression Analysis

Both Bayesian univariate and multivariate logistic regression analyses were applied to the data to determine which factors were associated with TM use (question [Q]3) and TM willingness (Q4A), respectively. In total, 22 independent variables were considered for each univariate regression analysis (Multimedia Appendix 1). The individuals who missed providing information on age or gender or answers to Q3 (467/492, 5.1%) and Q4A (454/492, 7.7%) were excluded. Otherwise, missing values (no answer) were considered as a new category for the univariate regression analysis. For instance, Q28, “assigning physician or rheumatologist,” previously had 2 categories and was revised to have 3 categories, “assigning physician,” “rheumatologist,” or “not answered”. For statistical analysis, all the categorical variables having >2 modalities, for example, “yes,” “no,” or “do not know,” were transformed into dummy or binary variables. For instance, Q21 was transformed into 3 dummy variables.

For each model, odds ratios (ORs) with 95% credible interval (CI) are presented. All the individual variables associated (positively or negatively) with TM use and TM willingness in the Bayesian univariate analysis were considered for analysis in the later Bayesian multivariate analysis (model selection) after variable selection. This variable selection was based on the region of practical equivalence (ROPE) percentage (ROPE%≤5) [16] and a subsequent selection based first on the variance inflation factor (VIF) [17]. Collinear covariates, with a VIF>2.5, were excluded in the multivariate models [18]. Finally, the determinants of TM use and TM willingness were identified through Bayesian model averaging (BMA) [19]. The “best” model (ie, model with the highest posterior probability) from BMA was detailed. All models were stratified by sex. In addition, determinant factors (question answers), defined as variables with a posterior probability of ≥10% with BMA, were identified and used to establish the profile of the individuals using or willing to use TM and the profile of the individuals not using or not willing to use TM using spider charts. For each determinant factor, the percentage of individuals who chose a specific answer was displayed on the spider chart. This percentage could range from 0 (the inner circular line, the closest to the center) if no individuals chose the specified answer for the considered question to 100 (the outer circular line, the farthest from the radar center) if all individuals answered the question with the specified answer. Green points and lines on the spider charts refer to the individuals who use or want to use TM, whereas red points and lines correspond to the individuals not willing to use or not using TM. For each question, there were 3 possible situations. When the green and red points overlapped (were similar), it meant that there was no difference between the individuals whether they were using TM or not or willing or not to use TM, that is, the proportion of similar answers was high. When the green point was higher (higher percentage) than the red point, it indicated that the individuals using or willing to use TM chose the specified answer more often than those not willing to use or not using TM, which meant that this factor (question) had a positive impact on TM use or TM willingness. Finally, when the green point was lower (lower percentage) than the red point, it indicated that the individuals willing to use or using TM chose the specified answer less often than the individuals not willing to use or not using TM, which meant that this factor (question) had a negative impact on TM use or TM willingness.

All statistical analyses were performed using R software (version 4.1.2, R Foundation for Statistical Computing) for Windows 10. The *tidyverse* package (version 1.3.2) was used [20]. VIFs were calculated using the *car* package (version 3.1-0) [21]. Bayesian estimation was performed using the *rstanarm* package (version 2.21.1) [22,23]. Weakly informative priors (default priors in *rstarnarm*) were used. The default priors in *rstanarm* 2.21.1 are designed to be weakly informative. The Bayesian model adds priors (independent by default) to the coefficients of the generalized linear model. The Bayesian estimation was performed via the Markov chain Monte Carlo Bernoulli model, with 4 randomly initialized Markov chains, each for 2000 iterations (including a warm-up period of 1000 iterations that is discarded). Posterior distributions were described using the *bayestestR* package (version 0.12.1) [24]. The selection of the “best” model through BMA was undertaken using the *BMA* package (version 3.18.15) [25]. Regarding priors for BMA, we assumed that all candidate models were equally likely a priori (same prior weight). The spider charts were created using the *fmsb* package (version 0.7.3) [26].

### Ethics Approval

Primary data collection was conducted in compliance with the current data protection regulations of the General Data Protection Regulation [27] and the Helsinki Declaration. All study participants were informed about the research project and provided written informed consent. Data were anonymized before analysis. The ethics committee of the Theodor Fontane Medical School in Brandenburg stated that no written consent was necessary owing to the noninterventional study design, which also applies to the secondary analysis.

## Results

### Population Characteristics

A total of 94.9% (467/492) and 92.3% (454/492) of individuals were selected for the analysis of TM use and TM willingness, respectively. Most participants (247/454, 54.4%) were female. Most individuals were GPs (408/454, 89.9%) and were aged between 51 and 60 years (215/454, 47.4%). Although most individuals were not using TM (344/454, 75.8%), two-thirds (282/454, 62.1%) were willing to use it in the future.

### Bayesian Univariate Logistic Regression Analysis

**Figure 1:**
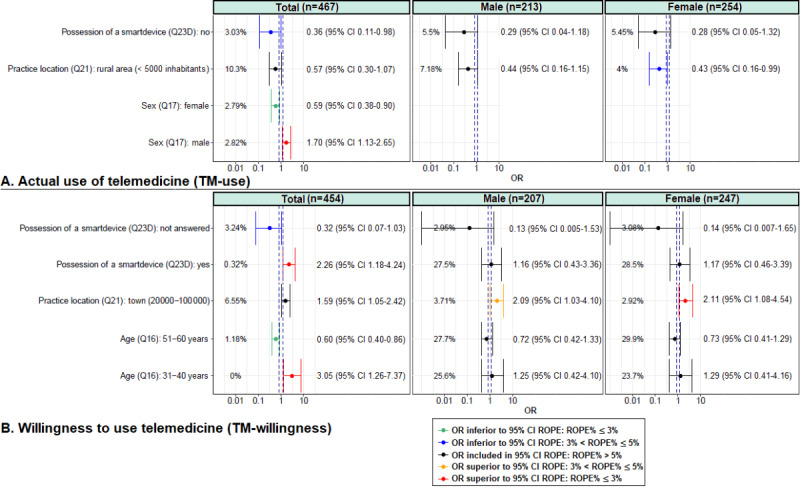
Bayesian univariate logistic regression—Relationship between the actual use of telemedicine (TM use) or willingness to use telemedicine (TM willingness) and sociodemographic factors. The percentage indicates the region of practical equivalence (ROPE) percentage, that is, the probability that the considered credible factor values are not negligible. The dashed lines indicate the 95% credible interval (CI) of the ROPE. OR: odds ratio; Q: question.

Only significant results are presented in the main text, but all the results can be found in the Multimedia Appendices 1-5 and Figures S1-S4 in Multimedia Appendix 6. A total of 26 questions were answered (83 answers) and analyzed using the univariate logistic regression analysis. Out of 83 variables, 36 (43%) and 34 (41%) variables were found to be positively or negatively associated (ROPE%≤5%) with TM use and TM willingness, respectively (Multimedia Appendix 2). Regarding sociodemographic factors (Figure 1), not owning a smart device (OR 0.36, 95% CI 0.11-0.99; ROPE%=3.0); being female (OR 0.59, 95% CI 0.38-0.90; ROPE%=2.8); and being female with a practice located in rural area (<5000 inhabitants; OR 0.43, 95% CI 0.16-0.99; ROPE%=4.0) were negatively associated with TM use, whereas being aged between 51 and 60 years (OR 0.60, 95% CI 0.40-0.86; ROPE%=1.2) was negatively associated with TM willingness. By contrast, being male (OR 1.70, 95% CI 1.13-2.65; ROPE%=2.8) was positively associated with TM use, whereas owning a smart device (OR 2.26, 95% CI 1.18-4.24; ROPE%=0.3); being aged 31 to 40 years (OR 3.05 95% CI 1.26-7.37; ROPE%=0); and having a practice located in town (20,000-100,000 inhabitants) were positively associated with TM willingness. For more details, please refer to Figures S1 and S2 in Multimedia Appendix 6.

Regarding work characteristics, being a rheumatologist, working in a medical care center, and treating >1000 patients per quarter were positively associated with TM use, whereas treating <500 patients per quarter and being an assigning physician were negatively associated (Multimedia Appendix 2 and Figures S3 and S4 in Multimedia Appendix 6).

Regarding the opinion and knowledge about TM, having at least good TM knowledge, thinking that TM is suitable for exchange in rheumatology, wanting to exchange information with specialists via TM, and thinking that TM is at least rather important for current and future work were positively associated with both TM use and TM willingness (Multimedia Appendix 2 and Figures S3-S6 in Multimedia Appendix 6). By contrast, having poor or very poor TM knowledge and thinking that TM is not important at all for current and future work were both negatively associated with both TM use and TM willingness. Individuals willing to use TM were strongly and positively associated with TM use.

### BMA and Bayesian Multivariate Logistic Regression Analysis

**Figure 2:**
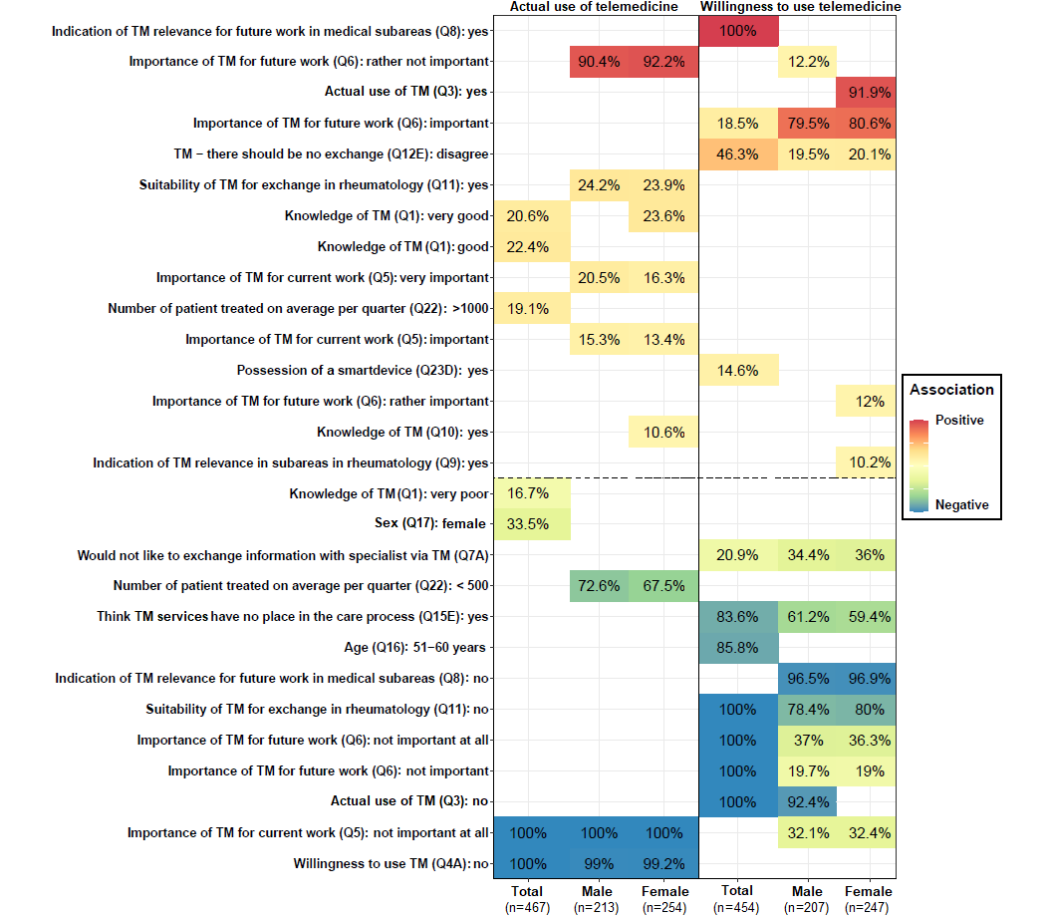
Determinants of the actual use of telemedicine (TM use) or willingness to use telemedicine (TM willingness) identified through the Bayesian model averaging analysis. A total of 28 answers from 16 questions were selected with Bayesian model averaging. The value in each cell corresponds to the posterior probability that the considered variable is nonzero (in percentage). Q: question.

A total of 6 BMA analyses were conducted, with 3 (both sexes, male, and female) for TM use and 3 for TM willingness. Figure 2 presents the determinants identified through BMA for the 6 analyses. Only variables with a posterior probability of ≥10% were considered determinant factors. A total of 16 answers were selected using BMA. Variables above the dashed horizontal line refer to factors positively associated with TM use or TM willingness (cells with color from light yellow to red). By contrast, variables under the dashed horizontal line refer to factors negatively associated with TM use or TM willingness (cells with colors from light green to dark blue). The value in each cell corresponds to the posterior probability that the considered variable is nonzero (in percentage). Darker the color, the higher the posterior probability percentage.

Regarding TM use, a total of 13 determinant factors (13 answers from 8 questions) were identified. Being female, having very poor knowledge of TM, treating <500 patients per quarter, thinking that TM is not important at all for current work, and not being willing to use TM were negatively associated with TM use. By contrast, having good or very good knowledge of TM, thinking that TM is important or very important for current work and at least rather not important for future work, treating >1000 patients per quarter, and thinking that TM is suitable for exchange in rheumatology were positively associated with TM use.

Regarding TM willingness, a total of 17 determinant factors (17 answers from 11 questions) were identified. Not wanting to exchange information with specialists using TM, thinking TM services have no place in the care process, being aged 51 to 60 years, thinking that TM is not important for current and future work, and not currently using TM were negatively associated with TM willingness. By contrast, owning a smart device, thinking that TM is at least rather not important for future work, thinking that TM is relevant in subareas in rheumatology, and thinking that there should be exchange with TM were positively associated with TM willingness.

For more details about the BMA analysis, please refer to Multimedia Appendix 4, which synthesizes BMA results for the top 5 models, as well as to Figures S7-S11 in Multimedia Appendix 6 for TM use and for TM willingness, which represent all the variables considered (in the y-axis) for the full list of models selected (in the x-axis). Blue color indicates variables negatively associated with TM use or TM willingness, whereas red color indicates variables that are positively associated.

Results for the “best” model identified through BMA indicated that being female (OR 0.57, 95% CI 0.35-0.90; ROPE%=3.2); thinking that TM is not important at all for current work (OR 0.15, 95% CI 0.08-0.29; ROPE%=0); and not being willing to use TM (OR 0.22, 95% CI 0.10-0.38; ROPE%=0) were negatively associated with TM use for both sexes. When stratified by sex, it was found that treating <500 patients per quarter was negatively associated with TM use. Regarding TM willingness, being aged 51 to 60 years (OR 0.43, 95% CI 0.26-0.74; ROPE%=0); not using TM (OR 0.14, 95% CI 0.06-0.31; ROPE%=0); thinking that TM is not suitable for exchange in rheumatology (OR 0.13, 95% CI 0.05-0.35; ROPE%=0); and thinking that it is not important for future work (OR 0.13, 95% CI 0.05-0.35; ROPE%=0) were factors negatively associated with TM willingness for both sexes.

More details about the “best” models are available in Multimedia Appendix 5.

### Profile of TM Users or Individuals Willing to Use TM

**Figure 3:**
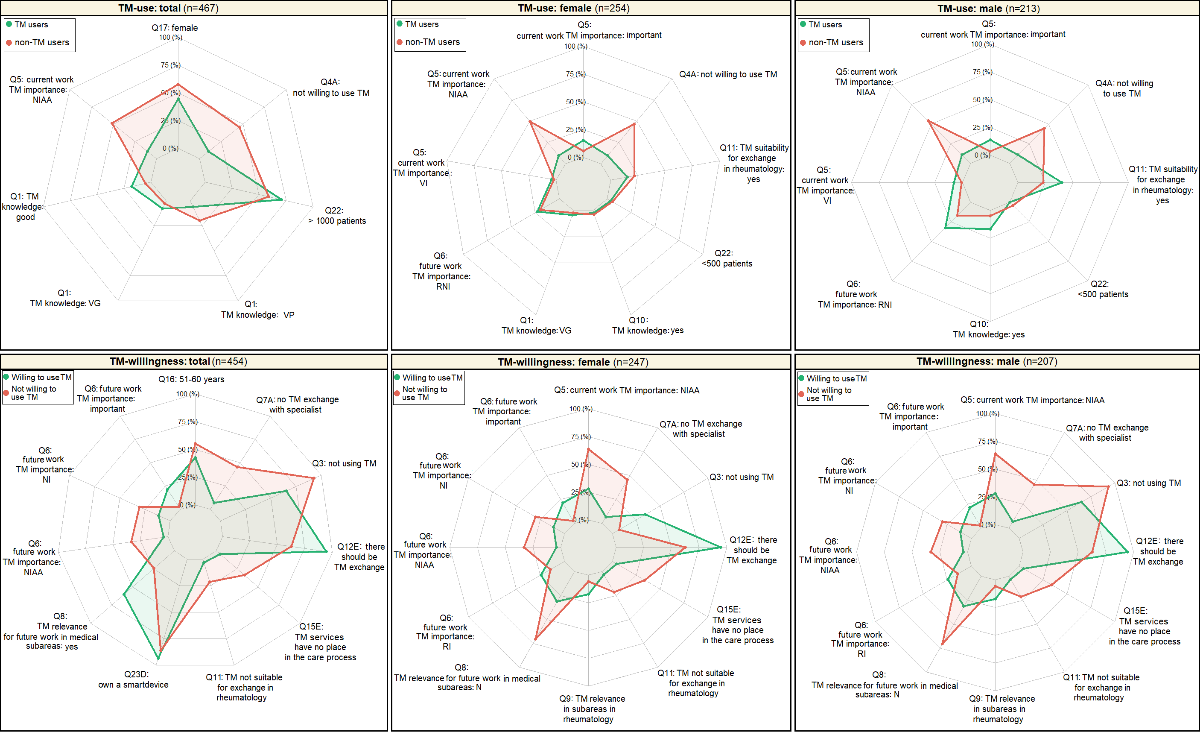
Profile of telemedicine (TM) users versus nonusers and individuals willing to use TM versus those not willing to use TM using Bayesian model averaging (BMA). Variables displayed on the spider or radar chart correspond to factors selected with BMA that had a posterior probability of ≥10%. Percentages refer to the percentage of individuals with the answer specified for each question. NI: not important; NIAA: not important at all; RI: rather important; RNI: rather not important; VG: very good; VI: very important; VP: very poor; TM willingness: willingness to use telemedicine; Q: question.

Determinant factors, defined as variables with a posterior probability of ≥10% with BMA, were identified and used to establish the profile of individuals using or willing to use TM and the profile of individuals not using or not willing to use TM. Figure 3 presents the profiles identified based on gender.

Regarding TM use, TM users more frequently had TM knowledge and treated, on average, more patients (>1000 patients per quarter) than non-TM users.

TM users were more often women, more often thought that TM is not important at all for current work, more frequently had very poor TM knowledge, and were less inclined to use TM compared with TM users.

Regarding TM willingness, the individuals who were willing to use TM owned a smart device and thought that there should be TM exchange more often than the individuals who were not willing to use TM. By contrast, the individuals not willing to use TM were more often aged 51 to 60 years and more frequently thought that TM is not suitable for exchange in rheumatology, is not important at all for current and future work, is not relevant for future work in medical subareas, and has no place in the care process. In addition, they used TM less often than the individuals who were willing to use TM.

## Discussion

### Overview

We performed a secondary analysis to identify factors associated with TM use and TM willingness on data collected as part of a cross-sectional, self-completed, and paper-based survey of German GPs and outpatient rheumatologists. The initial study [7] was conducted from September to November 2018, with the goal of exploring general acceptance, opportunities, and obstacles for the implementation of TM. The current secondary analysis was conducted to identify the most relevant factors affecting TM use and TM willingness to enable more effective TM strategies.

### Principal Findings

Regarding the factors associated with TM use, our results revealed that having good or very good knowledge of TM and treating >1000 patients per quarter were positively associated with TM use. By contrast, being female, having very poor knowledge of TM, treating <500 patients per quarter, not owning a smart device, working in a rural area, thinking that TM is not important at all for current work, and not being willing to use TM were negatively associated with TM use.

Regarding the factors associated with TM willingness, owning a smart device, thinking that TM is relevant in subareas in rheumatology, working in urban areas, and thinking that there should be exchange with TM were positively associated with TM willingness. By contrast, not wanting to exchange information with specialists using TM, thinking that TM services have no place in the care process, being aged 51 to 60 years, thinking that TM is not important for current and future work, and not currently using TM were negatively associated with TM willingness.

### Comparison With Prior Work

To the best of our knowledge, this is the first work analyzing specific factors influencing TM use and TM willingness among German GPs and rheumatologists. A major strength of this study lies in its ability to guide TM implementation strategies.

Our results underline the close connection between knowledge and technology use, as described by Paul Attewell [28]. According to his theory on technology diffusion and organizational learning, knowledge barriers—that is, the lack of knowledge about the technology and how this technology can be applied in an organizational setting—are in fact the reasons why technology diffusion remains low. Consistently, we found that having good or very good self-perceived knowledge of TM is positively associated with TM use, whereas having very poor knowledge is negatively associated with TM use. Similarly, a previous survey study identified the unawareness of suitable software solutions as the main factor that prevented rheumatologists from using electronic instead of paper-based questionnaires [29]. Concurringly, German rheumatologists were only aware of a fraction of the available rheumatology apps, limiting their use in clinical routine [30].

Tanriverdi and Iacono [31] extended Attewell’s theory to a multidimensional concept including the economic, organizational, and behavioral knowledge barriers that hamper the diffusion of TM. Our results support this multidimensionality. For instance, larger medical practices providing for more patients are more likely to use TM than smaller organizational units. Furthermore, in line with the results of Knörr et al [32], physicians in rural areas appear to use TM less frequently than physicians in urban areas, which seems counterintuitive and might also be due to the limited technical infrastructure in rural areas in Germany [8]. However, Vossen et al [33] reported a positive correlation between the traveling time to the treating rheumatologist and the willingness of German patients with rheumatoid arthritis to use video consultations.

In addition, the purchase of technology equipment, administration effort, and inadequate reimbursement (system) of TM services in Germany were identified as the main barriers to TM use in the primary analysis [7]. These barriers were later confirmed in a multiprofessional survey to impact TM use in other medical domains as well [34].

In line with the previous results reported by Alkureishi [35], our analysis results indicated a negative association between being female and TM use. We were surprised by this finding, as eHealth literacy was recently reported to be higher among women, both among health care professionals [36] and the overall German population [37]. Apparently, higher eHealth literacy does not translate directly into higher TM use. The reasons for the gender difference need to be specifically explored in further research, particularly as the proportion of women among physicians continues to increase in Germany [38]. Furthermore, the negative association between being aged 51 to 60 years and TM willingness is striking, as the average age of physicians in Germany is currently 54.2 years with an increasing trend [38]. This is linked to substantial concerns about increasing workforce shortage [5], which TM is actually intended to address [6,39]. However, a previous study on mobile health found no gender differences in patients with rheumatoid arthritis yet revealed a negative correlation with age [40]. Thus, the differences between the study findings may also be explained by specific TM approaches queried and terminology, which should be further researched.

### Implications

Because TM use is closely intertwined with physicians’ knowledge in this domain, we strongly support the integration of digital competencies into medical education and offering of dedicated training courses for physicians [41-43]. Continuous education in this area seems to be particularly important, as telemedical options continuously increase, including not only medical apps but also completely new procedures such as patient self-sampling. Health care professionals also seem concerned with an increasing workload due to increasing communication and transmitted information via TM [8]; education could help to implement the most successful TM strategies. As Tanriverdi and Iacono [31] discussed earlier, these training courses should also reflect on the multidimensionality of knowledge barriers by addressing the economic, organizational, and behavioral framework conditions of digital health implementation. Administrative, technical, and reimbursement requirements should be addressed first, as these have been reported as key barriers to the use of TM [7], just as they have recently been to the use of prescribed and regulated digital therapies in Germany [44].

Concomitantly, our data point to the importance of the organizational determinants of TM use. Although there are already numerous studies that point to the effectiveness of TM use [3], it remains unclear how TM needs to be integrated into organizational structures to ensure its effective and sustainable use in routine health care. Therefore, we recommend investigating the organizational and social factors of the implementation of TM and digital health in health care delivery.

Furthermore, our findings will inform private and public stakeholders on TM implementation. Public stakeholders, such as health policy makers, might use our findings to promote TM and upgrade infrastructure in rural areas. Specific target groups for incentive schemes could be female physicians aged 51 to 60 years in particular. Private stakeholders, such as TM companies or start-ups, might infer from our findings that health care professionals need low-threshold instructions on the use of their products. Finally, we recommend organizational and structural guidance, including setup, staff planning, billing of services, and administration, for the implementation of TM in routine health care delivery.

### Limitations

The primary data on which this analysis was based were collected in 2018 before the SARS-CoV-2 outbreak. Owing to the need to reduce physical contact and thus minimize the risk of infection, TM use initially received a major uptake in global health care delivery [13]. Hence, more physicians and likely other subgroups will have tried TM by now [23], which has led to an increased use and awareness of TM in routine practice. Nevertheless, recent studies suggest that even after the SARS-CoV-2 outbreak, the same barriers continue to prevent widespread TM adoption [9,10,35,44,45]. However, a replication of the initial survey is essential to identify whether and how the identified factors have changed in the surveyed target group. Thus, the results from our study represent a baseline to future studies that would investigate the change in TM experience and perceptions due to the SARS-CoV-2 pandemic.

Apart from the aforementioned shortcomings, the limitations of the primary data still apply [7]. Only a relatively small proportion (44/454, 9.7%) of the survey sample are rheumatologists, which accounts for 7% of all of the rheumatologists in outpatient care in Germany [46]. Although the survey was directed at physicians from all over Germany, it was primarily physicians from Brandenburg who participated because of the recruitment strategy. We suspect a high potential of self-selection and nonresponse bias. Health care professionals in inpatient care as well as other professions involved in rheumatology care (eg, nurses) were not included in the survey. Furthermore, our results cover the perspectives of German physicians only. Their acceptance of TM might be strongly influenced by the specifics and policy drivers of the German health system. Previous studies reported [8,45] weak remuneration, high bureaucracy, and a lack of digital infrastructure to hamper TM use in Germany. Owing to these influences, the transferability of our results to other countries and health care systems may be limited. Finally, physician engagement is an important factor in the adoption of telehealth into routine care delivery, but it represents only one side of the coin. The patient perspective and TM willingness represent the other side that needs to be investigated as a priority.

Regarding the statistical analysis, we used a Bayesian approach to conduct the secondary analysis of the aforementioned survey. A practical limitation of the Bayesian approach is that it requires the specification of prior distributions both on the parameters of each model and on the distribution of the models themselves. Because we had no a priori assumption, we used weakly informative priors. Choosing another prior distribution may have had substantial influence on the outcome [47,48]. Regarding the variable and model selections, a 3-step approach was used. First, all the individual variables associated (positively or negatively) with the use of or TM willingness in the Bayesian univariate analysis were selected based on the ROPE percentage (ROPE%≤5). Choosing a different ROPE percentage threshold may have yielded different results. Then, we performed a conservative selection based on the VIF (VIF≤2.5) to deal with potential variable multicollinearity. Finally, we used the remaining variables with BMA for model selection and identification of determinants. BMA was chosen in particular because it reduces overconfidence and is relatively robust against model misspecification [47,49-51]. Markov chain Monte Carlo was used to deal with the intractable computational challenge of BMA that comes from the candidate model enumeration [52].

### Conclusions

TM use is intertwined with health care professionals' knowledge of TM. Limited knowledge restricts the implementation of TM in rheumatology care. Dedicated education courses could provide the necessary knowledge and improve TM uptake. These courses need to reflect on the multidimensionality of knowledge barriers by addressing the economic, organizational, and behavioral framework conditions of TM implementation.

TM willingness is associated with age and practice location, and incentive programs for advanced physicians practicing in rural areas have the potential to increase the implementation of TM in standard care.

## Appendix

Multimedia Appendix 1 Regression analysis—variables.

Multimedia Appendix 2 List of all the variables positively and negatively associated (region of practical equivalence≤5%) with the actual use of telemedicine use and willingness to use telemedicine in the Bayesian univariate logistic regression analysis.

Multimedia Appendix 3 Bayesian univariate logistic regression analysis results.

Multimedia Appendix 4 Bayesian model averaging results.

Multimedia Appendix 5 Bayesian multivariate logistic regression analysis results for the best model.

Multimedia Appendix 6 Bayesian univariate logistic regression figures.

## Abbreviations

BMA: Bayesian model averaging
CI: credible interval
GP: general practitioner
OR: odds ratio
ROPE: region of practical equivalence
TM use: actual use of telemedicine
TM willingness: willingness to use telemedicine
TM: telemedicine
VIF: variance inflation factor
Q: question

## References

[1] (2018). WMA Statement on the Ethics of Telemedicine. World Medical Association.

[2] Piga M, Cangemi I, Mathieu A, Cauli A (2017). Telemedicine for patients with rheumatic diseases: systematic review and proposal for research agenda. Semin Arthritis Rheum.

[3] McDougall JA, Ferucci ED, Glover J, Fraenkel L (2017). Telerheumatology: a systematic review. Arthritis Care Res (Hoboken).

[4] Sebbag E, Felten R, Sagez F, Sibilia J, Devilliers H, Arnaud L (2019). The world-wide burden of musculoskeletal diseases: a systematic analysis of the World Health Organization Burden of Diseases Database. Ann Rheum Dis.

[5] Al Maini M, Adelowo F, Al Saleh J, Al Weshahi Y, Burmester G, Cutolo M, Flood J, March L, McDonald-Blumer H, Pile K, Pineda C, Thorne C, Kvien TK (2015). The global challenges and opportunities in the practice of rheumatology: white paper by the World Forum on Rheumatic and Musculoskeletal Diseases. Clin Rheumatol.

[6] Ward IM, Schmidt TW, Lappan C, Battafarano DF (2016). How critical is tele-medicine to the rheumatology workforce?. Arthritis Care Res (Hoboken).

[7] Muehlensiepen F, Knitza J, Marquardt W, Engler J, Hueber A, Welcker M (2021). Acceptance of telerheumatology by rheumatologists and general practitioners in Germany: nationwide cross-sectional survey study. J Med Internet Res.

[8] Muehlensiepen F, Knitza J, Marquardt W, May S, Krusche M, Hueber A, Schwarz J, Vuillerme N, Heinze M, Welcker M (2021). Opportunities and barriers of telemedicine in rheumatology: a participatory, mixed-methods study. Int J Environ Res Public Health.

[9] Bruch D, Muehlensiepen F, Alexandrov A, Konstantinova Y, Voß K, Ronckers C, Neugebauer E, May S (2021). The impact of the COVID-19 pandemic on professional practice and patient volume in medical practices: a survey among German physicians and psychotherapists. Z Evid Fortbild Qual Gesundhwes.

[10] Richter JG, Chehab G, Reiter J, Aries P, Muehlensiepen F, Welcker M, Acar H, Voormann A, Schneider M, Specker C (2022). Evaluation of the use of video consultation in German rheumatology care before and during the COVID-19 pandemic. Front Med (forthcoming).

[11] Portnoy J, Waller M, Elliott T (2020). Telemedicine in the era of COVID-19. J Allergy Clin Immunol Pract.

[12] Smith AC, Thomas E, Snoswell CL, Haydon H, Mehrotra A, Clemensen J, Caffery LJ (2020). Telehealth for global emergencies: implications for coronavirus disease 2019 (COVID-19). J Telemed Telecare.

[13] Omboni S, Padwal RS, Alessa T, Benczúr B, Green BB, Hubbard I, Kario K, Khan NA, Konradi A, Logan AG, Lu Y, Mars M, McManus RJ, Melville S, Neumann CL, Parati G, Renna NF, Ryvlin P, Saner H, Schutte AE, Wang J (2022). The worldwide impact of telemedicine during COVID-19: current evidence and recommendations for the future. Connect Health.

[14] El Aoufy K, Melis MR, Bellando Randone S, Blagojevic J, Bartoli F, Fiori G, Nacci F, Conforti ML, Cometi L, Bruni C, Orlandi M, Moggi-Pignone A, Rasero L, Guiducci S, Matucci-Cerinic M (2022). The positive side of the coin: Sars-Cov-2 pandemic has taught us how much Telemedicine is useful as standard of care procedure in real life. Clin Rheumatol.

[15] (2021). Veränderung der vertragsärztlichen Leistungsinanspruchnahme während der COVID-Krise. Zentralinstitut für die kassenärztliche Versorgung in Deutschland.

[16] Kruschke JK (2018). Rejecting or accepting parameter values in Bayesian estimation. Adv Methods Pract Psychol Sci.

[17] Midi H, Sarkar SK, Rana S (2010). Collinearity diagnostics of binary logistic regression model. J Interdisciplinary Math.

[18] Zuur AF, Iono EN, Elphick CS (2010). A protocol for data exploration to avoid common statistical problems. Methods Ecol Evol.

[19] Depaoli S, Lai K, Yang Y (2021). Bayesian model averaging as an alternative to model selection for multilevel models. Multivariate Behav Res.

[20] Brilleman S, Crowther M, Moreno-Betancur M, Buros Novik J, Wolfe R (2018). Joint longitudinal and time-to-event models via Stan. GitHub.

[21] Wickham H, Averick M, Bryan J, Chang W, McGowan LD, François R, Grolemund G, Hayes A, Henry L, Hester J, Kuhn M, Pedersen T, Miller E, Bache S, Müller K, Ooms J, Robinson D, Seidel DP, Spinu V, Takahashi K, Vaughan D, Wilke C, Woo K, Yutani H (2019). Welcome to the Tidyverse. J Open Source Softw.

[22] Fox J, Weisberg S (2019). An R companion to applied regression. 3rd edition. Sage Publications.

[23] Goodrich B, Gabry J, Ali I, Brilleman S (2020). rstanarm: Bayesian applied regression modeling via Stan. R package version 2.21.1.

[24] Makowski D, Ben-Shachar MS, Lüdecke D (2019). bayestestR: describing effects and their uncertainty, existence and significance within the Bayesian framework. J Open Source Softw.

[25] Raftery A, Hoeting J, Volinsky C, Painter I, Yeung KY (2021). BMA: Bayesian Model Averaging. Package for Bayesian model averaging and variable selection for linear models, generalized linear models and survival models (cox regression). Version 3.18.15. GitHub.

[26] Nakazawa M (2022). fmsb: Functions for Medical Statistics Book with some Demographic Data. The Comprehensive R Archive Network.

[27] The European Parliament and the Council of the European Union Consolidated text: Regulation (EU) 2016/679 of the European Parliament and of the Council of 27 April 2016 on the protection of natural persons with regard to the processing of personal data and on the free movement of such data, and repealing Directive 95/46/EC (General Data Protection Regulation) (Text with EEA relevance). EUR-Lex.

[28] Attewell P (1992). Technology diffusion and organizational learning: the case of business computing. Organ Sci.

[29] Krusche M, Klemm P, Grahammer M, Mucke J, Vossen D, Kleyer A, Sewerin P, Knitza J (2020). Acceptance, usage, and barriers of electronic patient-reported outcomes among German rheumatologists: survey study. JMIR Mhealth Uhealth.

[30] Knitza J, Vossen D, Geffken I, Krusche M, Meyer M, Sewerin P, Kleyer A, Hueber AJ, Arbeitskreis Junge Rheumatologen (2019). Use of medical apps and online platforms among German rheumatologists : results of the 2016 and 2018 DGRh conference surveys and research conducted by rheumadocs. Z Rheumatol.

[31] Tanriverdi H, Iacono CS (1999). Diffusion of telemedicine: a knowledge barrier perspective. Telemed J.

[32] Knörr V, Dini L, Gunkel S, Hoffmann J, Mause L, Ohnhäuser T, Stöcker A, Scholten N (2022). Use of telemedicine in the outpatient sector during the COVID-19 pandemic: a cross-sectional survey of German physicians. BMC Prim Care.

[33] Vossen D, Knitza J, Klemm P, Haase I, Mucke J, Kernder A, Meyer M, Kleyer A, Sewerin P, Bendzuck G, Eis S, Krusche M, Morf H (2021). Acceptance of video consultation among patients with inflammatory rheumatic diseases depends on gender and location-results of an online survey among patients and physicians. Z Rheumatol (forthcoming).

[34] Peine A, Paffenholz P, Martin L, Dohmen S, Marx G, Loosen SH (2020). Telemedicine in Germany during the COVID-19 pandemic: multi-professional national survey. J Med Internet Res.

[35] Alkureishi MA, Choo ZY, Lenti G, Castaneda J, Zhu M, Nunes K, Weyer G, Oyler J, Shah S, Lee WW (2021). Clinician perspectives on telemedicine: observational cross-sectional study. JMIR Hum Factors.

[36] Shiferaw KB, Mehari EA (2019). Internet use and eHealth literacy among health-care professionals in a resource limited setting: a cross-sectional survey. Adv Med Educ Pract.

[37] (2022). TechnikRadar 2022. Koerber Stiftung.

[38] Health Data. Kassenärztliche Bundesvereinigung.

[39] Ravindran V, Kataria S (2019). Digital health in rheumatology. Ann Rheum Dis.

[40] Knitza J, Simon D, Lambrecht A, Raab C, Tascilar K, Hagen M, Kleyer A, Bayat S, Derungs A, Amft O, Schett G, Hueber AJ (2020). Mobile health usage, preferences, barriers, and eHealth literacy in rheumatology: patient survey study. JMIR Mhealth Uhealth.

[41] Khurana MP, Raaschou-Pedersen DE, Kurtzhals J, Bardram JE, Ostrowski SR, Bundgaard JS (2022). Digital health competencies in medical school education: a scoping review and Delphi method study. BMC Med Educ.

[42] Yeoh SA, Young K, Putman M, Sattui S, Conway R, Graef E, Kilian A, Konig M, Sparks J, Ugarte-Gil M, Upton L, Berenbaum F, Bhana S, Costello W, Hausmann J, Machado P, Robinson P, Sirotich E, Sufka P, Yazdany J, Liew J, Grainger R, Wallace Z, Jayatilleke A, Global Rheumatology Alliance (2022). Rapid adoption of telemedicine in rheumatology care during the COVID-19 pandemic highlights training and supervision concerns among rheumatology trainees. ACR Open Rheumatol.

[43] Almufleh A, Lee C, Tsang MY, Gin K, Tsang TS, Nair P (2021). The need for telemedicine integration into adult cardiology training curricula in Canada. Can J Cardiol.

[44] Dahlhausen F, Zinner M, Bieske L, Ehlers JP, Boehme P, Fehring L (2022). There's an app for that, but nobody's using it: insights on improving patient access and adherence to digital therapeutics in Germany. Digit Health.

[45] Mühlensiepen F, Kurkowski S, Krusche M, Mucke J, Prill R, Heinze M, Welcker M, Schulze-Koops H, Vuillerme N, Schett G, Knitza J (2021). Digital health transition in rheumatology: a qualitative study. Int J Environ Res Public Health.

[46] Froschauer S, Muth T, Bredow L, Feist E, Heinemann-Dammann SP, Zinke S, Fiehn C (2021). Versorgungsatlas rheumatologie : ansätze und konzepte zur verbesserung der versorgung in der ambulanten rheumatologie. Z Rheumatol.

[47] Hinne M, Gronau QF, van den Bergh D, Wagenmakers EJ (2020). A conceptual introduction to Bayesian model averaging. Adv Methods Pract Psychol Sci.

[48] Wagenmakers EJ, Marsman M, Jamil T, Ly A, Verhagen J, Love J, Selker R, Gronau QF, Šmíra M, Epskamp S, Matzke D, Rouder JN, Morey RD (2018). Bayesian inference for psychology. Part I: theoretical advantages and practical ramifications. Psychon Bull Rev.

[49] Genell A, Nemes S, Steineck G, Dickman PW (2010). Model selection in medical research: a simulation study comparing Bayesian model averaging and stepwise regression. BMC Med Res Methodol.

[50] Mu Y, See I, Edwards JR (2019). Bayesian model averaging: improved variable selection for matched case-control studies. Epidemiol Biostat Public Health.

[51] Wang D, Zhang W, Bakhai A (2004). Comparison of Bayesian model averaging and stepwise methods for model selection in logistic regression. Stat Med.

[52] Lu Z, Lou W (2021). Bayesian approaches to variable selection: a comparative study from practical perspectives. Int J Biostat.

