# Supplementary material for: Factors Associated With Telemedicine Use Among German General Practitioners and Rheumatologists: Secondary Analysis of Data From a Nationwide Survey
Source: J Med Internet Res. 2022 Nov 30;24(11):e40304. doi: 10.2196/40304 (PMC9752470; doi:10.2196/40304)
Supplement: Multimedia Appendix 1 [file jmir_v24i11e40304_app1.pdf]

Multimedia Appendix 1: Table. Regression analysis - variables.

**Dependent variables**	**Modality**
Q3: “Do you use telemedicine?”	2 categories: yes or no
Q4A: “Would you like to use telemedicine?”	2 categories: yes or no
**Independent variables**	
Q1: “How would you rate your knowledge of telemedicine?”	7 categories: very good, good, satisfactory, unsatisfactory, poor, very poor, not answered
Q2: “Are you familiar with the following concept? (telemedicine)”	3 categories: yes, no, not answered
Q3: “Do you use telemedicine?” (when Q4A is considered as the dependent variable)	3 categories: yes, no, not answered
Q4A: “Would you like to use telemedicine?” (when Q3 is considered as the dependent variable)	3 categories: yes, no, not answered
Q5: “When you consider your current work situation, how do you assess the importance of telemedicine for your work?”	7 categories: very important, important, rather important, rather not important, not important, not important at all, not answered
Q6: “When you consider your future work situation, how do you assess the importance of telemedicine for your work?”	7 categories: very important, important, rather important, rather not important, not important, not important at all, not answered
Q7A: “With which specialist group would you like to exchange information via telemedicine? (none)”	3 categories: yes, no, does not apply
Q8: “Can you indicate medical subareas in which you consider telemedicine to be particularly relevant for your future work?”	3 categories: yes, no, not answered
Q9: “Can you indicate subareas in rheumatology care in which you consider telemedicine to be particularly relevant?”	4 categories: yes, no, does not apply, not answered
Q10: “Do you know of telemedicine approaches specifically for rheumatology?”	4 categories: yes, no, does not apply, not answered
Q11: “Do you think telemedicine is a suitable tool for exchange in rheumatology?”	4 categories: yes, no, does not apply, not answered
Q15E: “At what point in the care process do you think the use of telemedicine services is appropriate? (none)”	3 categories: yes, no, not answered
Q16: “Age”	5 categories: < 30 years, 31-40 years, 41-50 years, 51-60 years, > 61 years
Q17: “Sex”	2 categories: female or male
Q18: “Specialty”	3 categories: GP, rheumatology, not answered
Q19: “Type of medical practice”	5 categories: single practice, joint practice, medical care center, others, not answered
Q20: “Are you an employed physician”	3 categories: yes, no, not answered
Q21: “How would you classify your practice location?”	5 categories: city (>100000 inhabitants), town (20000-100000 inhabitants), provincial town (5000-20000 inhabitants), rural area (<5000), not answered
Q22: “How many patients do in total do you treat on average per quarter?”	4 categories: < 500, 500-1000, >1000, not answered
Q23D: “Which of the following devices do you possess? (None)”	3 categories: yes, no, not answered
Q28: “Assigning physician / Rheumatologist”	3 categories: assigning physician, rheumatologist, not answered
Q30: “Federal State of Brandenburg / German reference Group”	2 categories: Brandenburg, reference group

Note: There was a total of 26 questions with 83 answers (modalities) that were considered for analysis.
