# Supplementary material for: Factors Associated With Telemedicine Use Among German General Practitioners and Rheumatologists: Secondary Analysis of Data From a Nationwide Survey
Source: J Med Internet Res. 2022 Nov 30;24(11):e40304. doi: 10.2196/40304 (PMC9752470; doi:10.2196/40304)
Supplement: Multimedia Appendix 2 [file jmir_v24i11e40304_app2.pdf]

Multimedia Appendix 2: Table. List of all the variables positively and negatively associated (region of practical equivalence≤5%) with the actual use of telemedicine use and willingness to use telemedicine in the Bayesian univariate logistic regression analysis.

Factor/Variable	TM-use		TM-willingness	
	Association	Sex	Association	Sex
Knowledge of TM (Q1): very good	Positive	Total		
Knowledge of TM (Q1): good	Positive	Total, female, male	Positive	Total, female, male
Knowledge of TM (Q1): poor	Negative	Total	Negative	Female, male
Knowledge of TM (Q1): very poor	Negative	Total, female, male		
Knowledge of TM concept (Q2): no			Negative	Total
Actual use of TM (Q3): no			Negative	Total, female, male
Actual use of TM (Q3): yes			Positive	Total, female, male
Willingness to use TM (Q4A): no	Negative	Total, female, male		
Willingness to use TM (Q4A): not answered*	Positive	Total, female, male		
Willingness to use TM (Q4A): yes	Positive	Total, female, male		
Importance of TM for current work (Q5): very important	Positive	Total, female, male	Positive	Female, male
Importance of TM for current work (Q5): important	Positive	Total, female, male	Positive	Total
Importance of TM for current work (Q5): rather important	Positive	Total, female, male	Positive	Total, female, male
Importance of TM for current work (Q5): rather not important	Positive	Total, female, male	Positive	Total
Importance of TM for current work (Q5): not important at all	Negative	Total, female, male	Negative	Total, female, male
Importance of TM for future work (Q6): very important	Positive	Female, male	Positive	Total, female, male
Importance of TM for future work (Q6): important	Positive	Total, female, male	Positive	Total, female, male
Importance of TM for future work (Q6): rather important			Positive	Total, female, male
Importance of TM for future work (Q6): rather not important	Positive	Female, male	Positive	Total
Importance of TM for future work (Q6): not important	Negative	Total, female, male	Negative	Total, female, male
Importance of TM for future work (Q6): not important at all	Negative	Total, female, male	Negative	Total, female, male
Would like to exchange information with specialist via TM (Q7A)*	Positive	Total, female, male	Positive	Total, female, male
Would not like to exchange information with specialist via TM (Q7A)	Negative	Total, female, male	Negative	Total, female, male
Indication of TM relevance for future work in medical subareas (Q8): no	Negative	Total, female, male	Negative	Total, female, male
Indication of TM relevance for future work in medical subareas (Q8): yes	Positive	Total	Positive	Total, female, male
Indication of TM relevance in subareas in rheumatology (Q9): no			Negative	Total, female, male
Indication of TM relevance in subareas in rheumatology (Q9): yes	Positive	Female, male	Positive	Total, female, male
Knowledge of TM (Q10): yes	Positive	Total, female, male	Positive	Total, female, male
Suitability of TM for exchange in rheumatology (Q11): no			Negative	Total, female, male
Suitability of TM for exchange in rheumatology (Q11): yes	Positive	Female, male	Positive	Total, female, male
TM - there should be no exchange (Q12E): agree	Negative	Total, female, male	Negative	Total, female, male
TM - there should be no exchange (Q12E): disagree	Positive	Total, female, male	Positive	Total, female, male
Think TM services have no place in the care process (Q15E): yes			Negative	Total, female, male
Think TM services have no place in the care process (Q15E): no	Positive	Female, male	Positive	Total, female, male
Age (Q16): 31-40 years			Positive	Total
Age (Q16): 51-60 years			Negative	Total
Sex (Q17): male*	Positive	Total		
Sex (Q17): female	Negative	Total		
Type of medical practice (Q19): medical care center	Positive	Female, male		
Employed physician (Q20): no			Negative	Total
Practice location (Q21): town (20000-100000 inhabitants)			Positive	Female, male
Practice location (Q21): rural area (<5000 inhabitants)	Negative	Female		
Number of patient treated on average per quarter (Q22): >1000	Positive	Total		
Number of patient treated on average per quarter (Q22): < 500	Negative	Female, male		
Possession of a smart device (Q23D): yes			Positive	Total
Possession of a smart device (Q23D): no	Negative	Total		
Physician type (Q28): assigning physician	Negative	Female, male		
Physician type (Q28): rheumatologist	Positive	Female, male		

Note: * collinear variables excluded (VIF > 2.5) for the multivariate analysis.
There was a total of 26 questions that were answered (83 answers) and analyzed with univariate logistic regression analysis.
