# Supplementary material for: Factors Associated With Telemedicine Use Among German General Practitioners and Rheumatologists: Secondary Analysis of Data From a Nationwide Survey
Source: J Med Internet Res. 2022 Nov 30;24(11):e40304. doi: 10.2196/40304 (PMC9752470; doi:10.2196/40304)
Supplement: Multimedia Appendix 6 [file jmir_v24i11e40304_app6.docx]

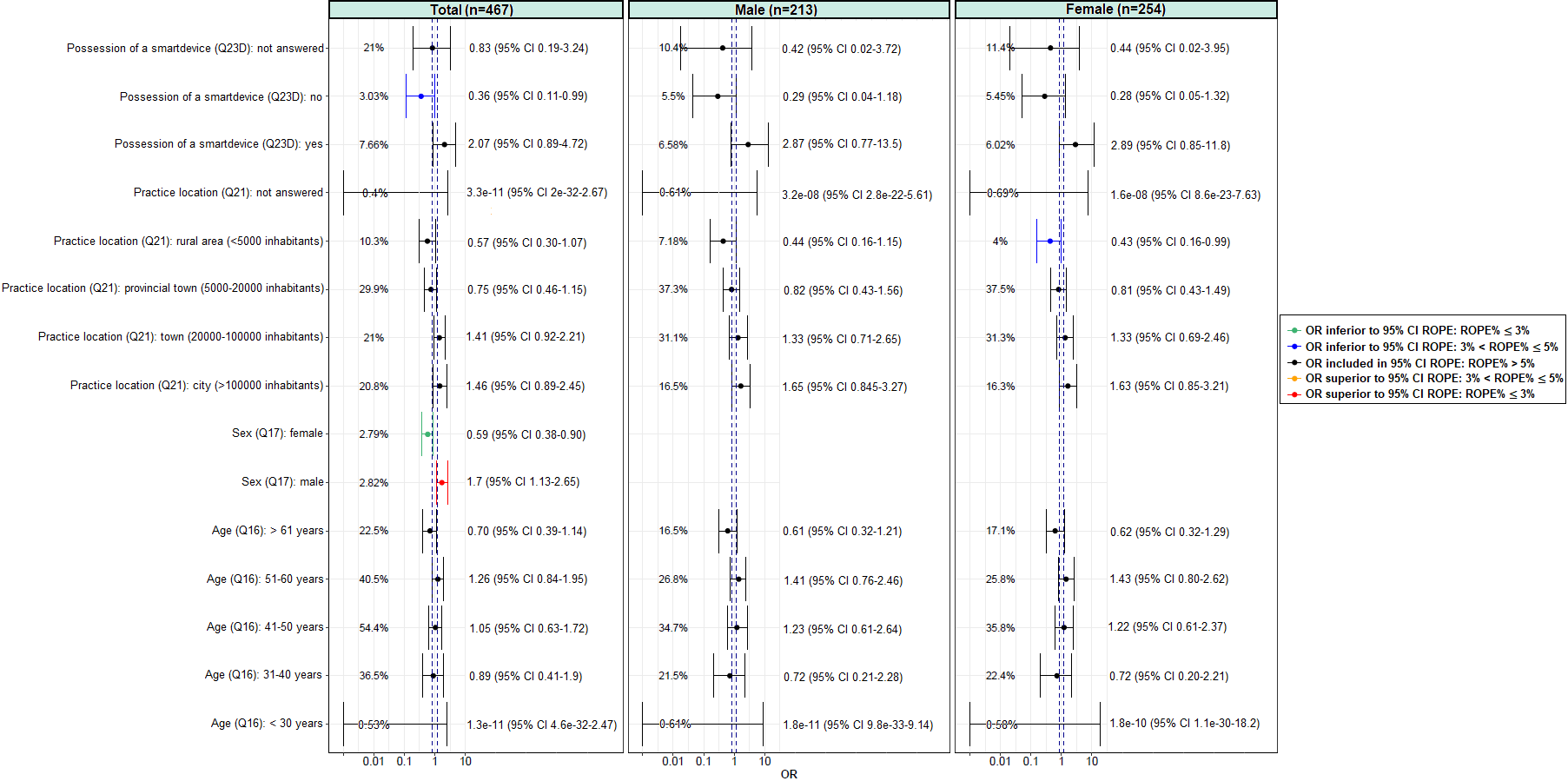


Figure S1. Bayesian univariate logistic regression: relationship between the actual use of telemedicine and sociodemographic factors.


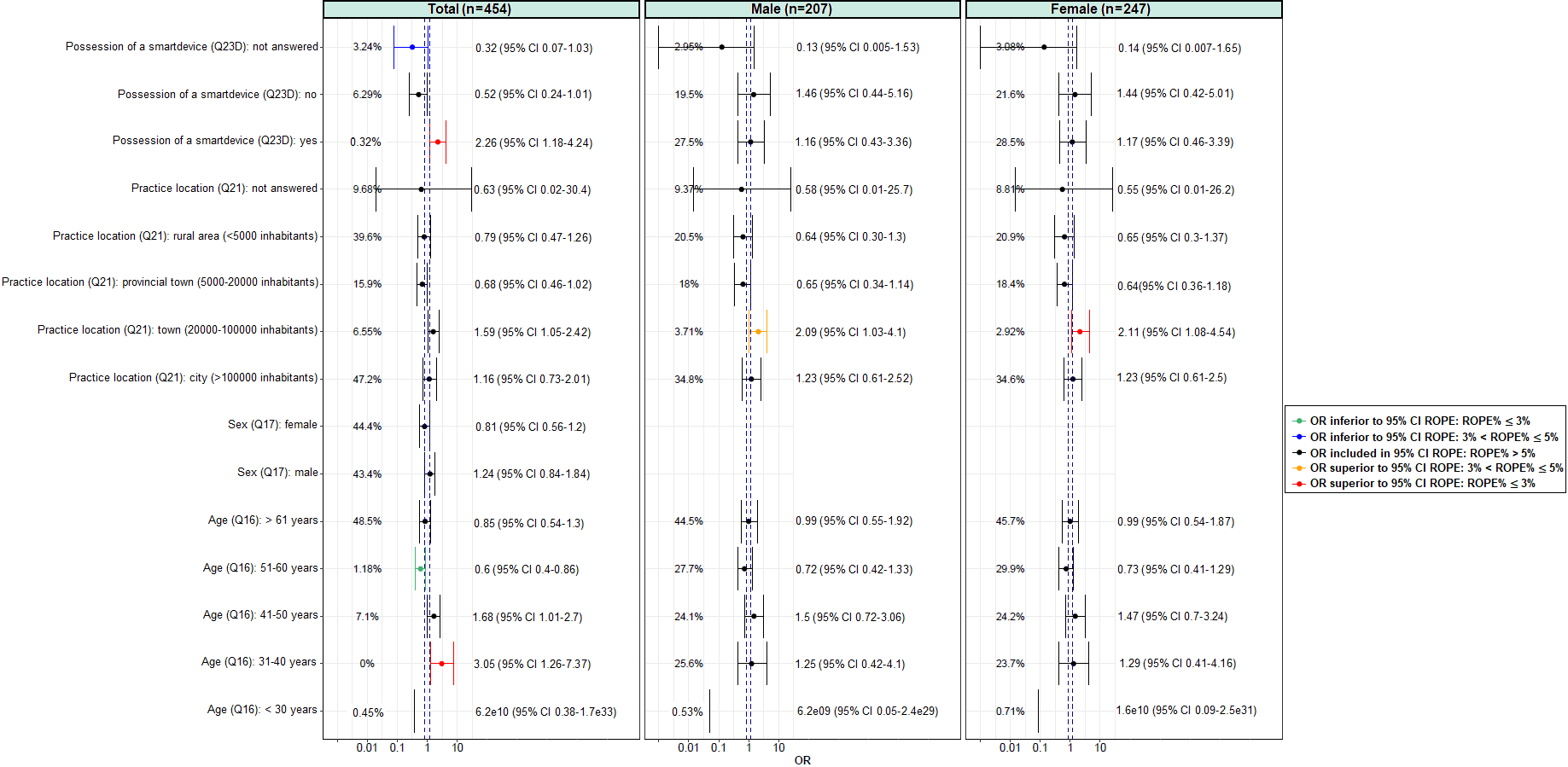


Figure S2. Bayesian univariate logistic regression: relationship between the willingness to use telemedicine and sociodemographic factors.


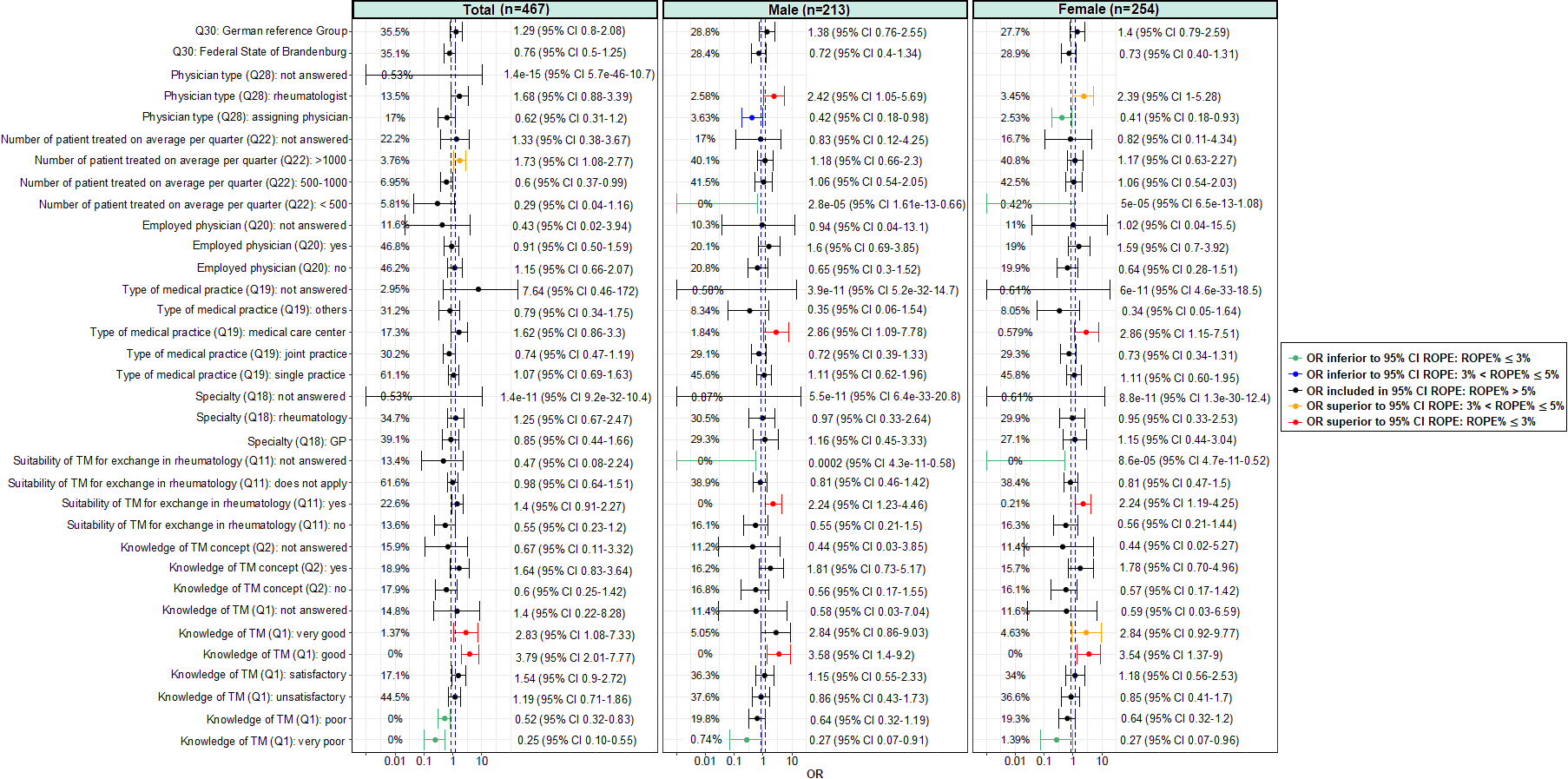


Figure S3. Bayesian univariate logistic regression: relationship between the actual use of telemedicine and work characteristics—part 1.


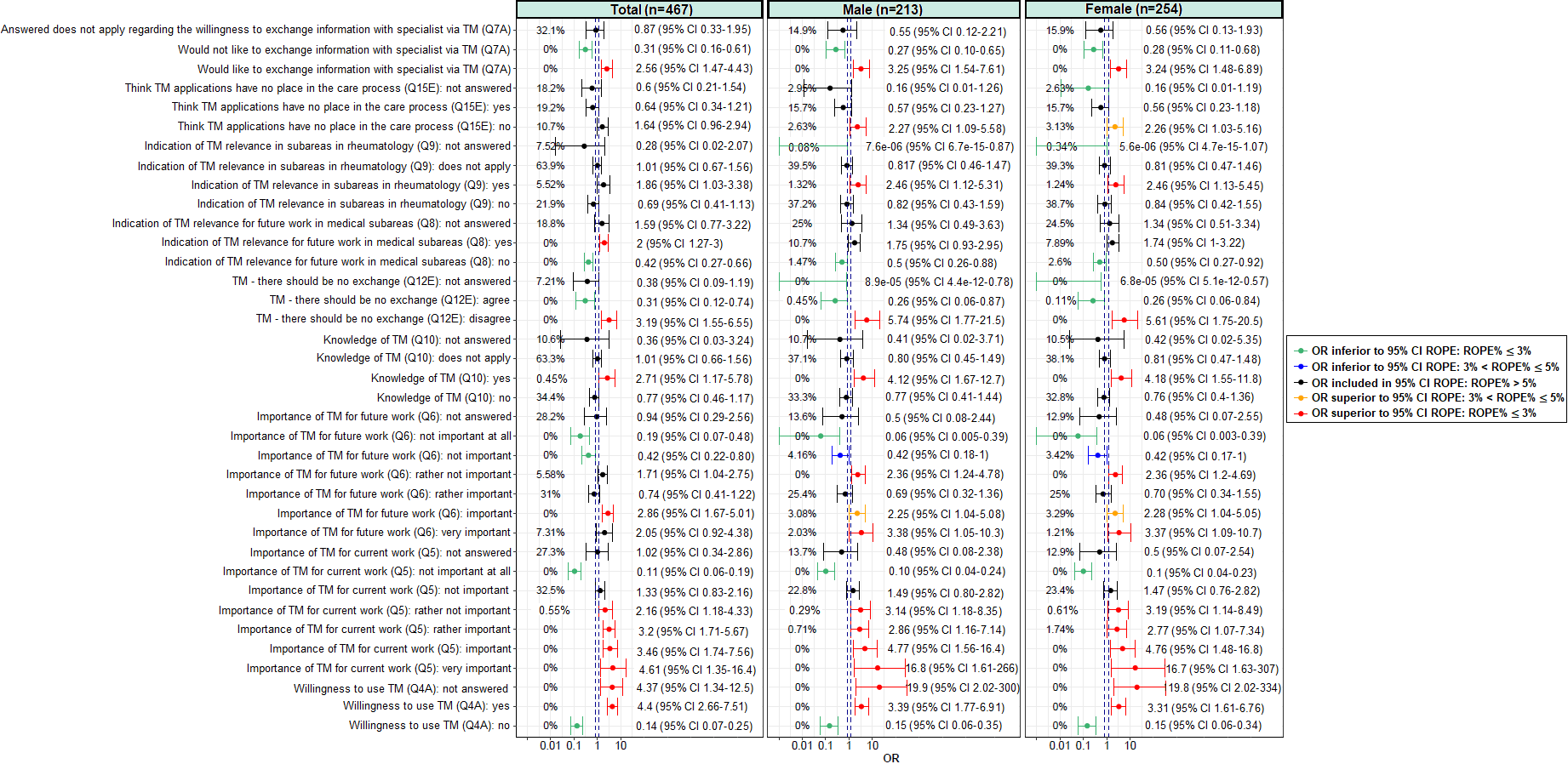


Figure S4. Bayesian univariate logistic regression: relationship between the actual use of telemedicine and work characteristics—part 2.


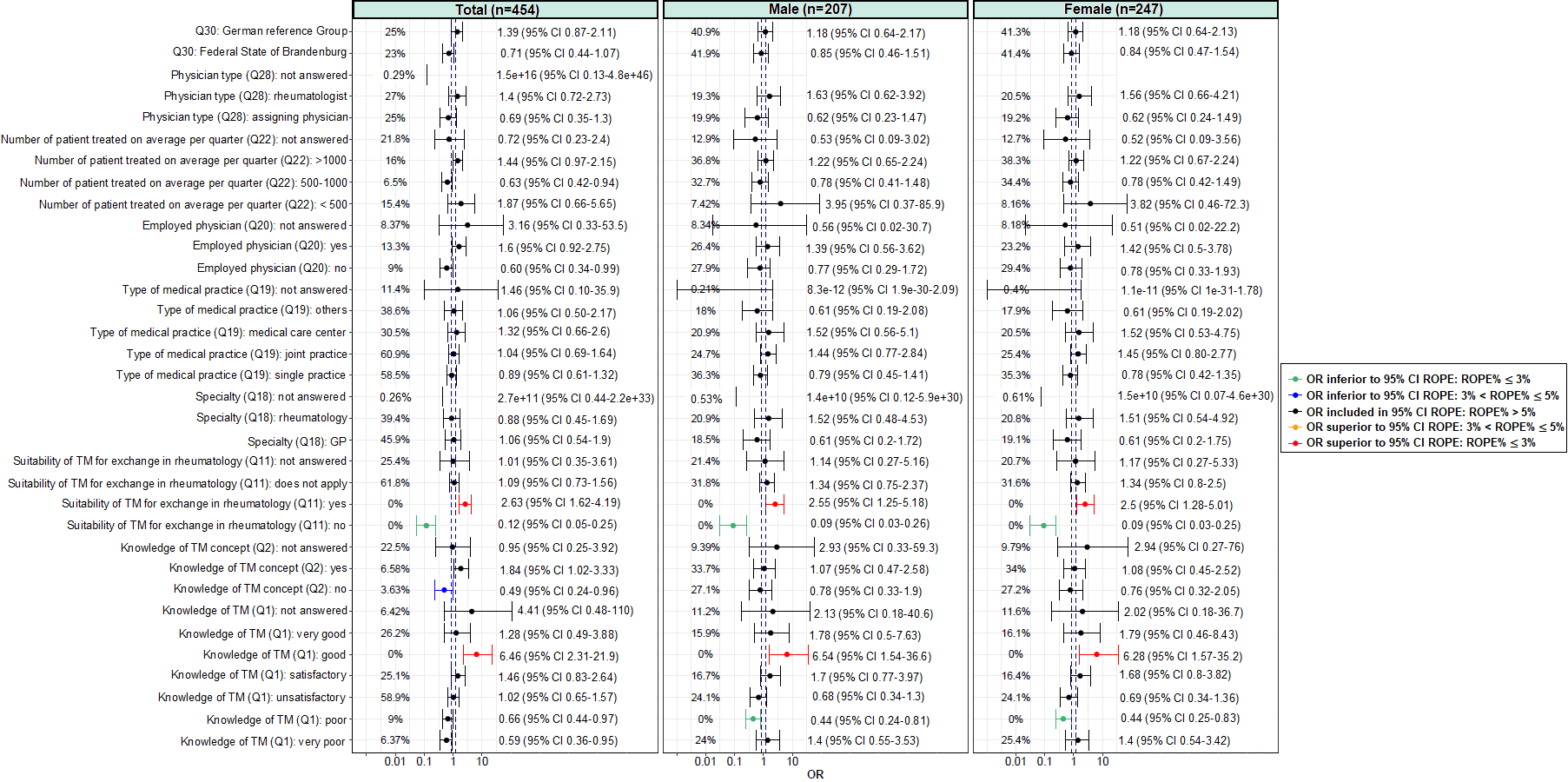


Figure S5. Bayesian univariate logistic regression: relationship between the willingness to use telemedicine and work characteristics—part 1.


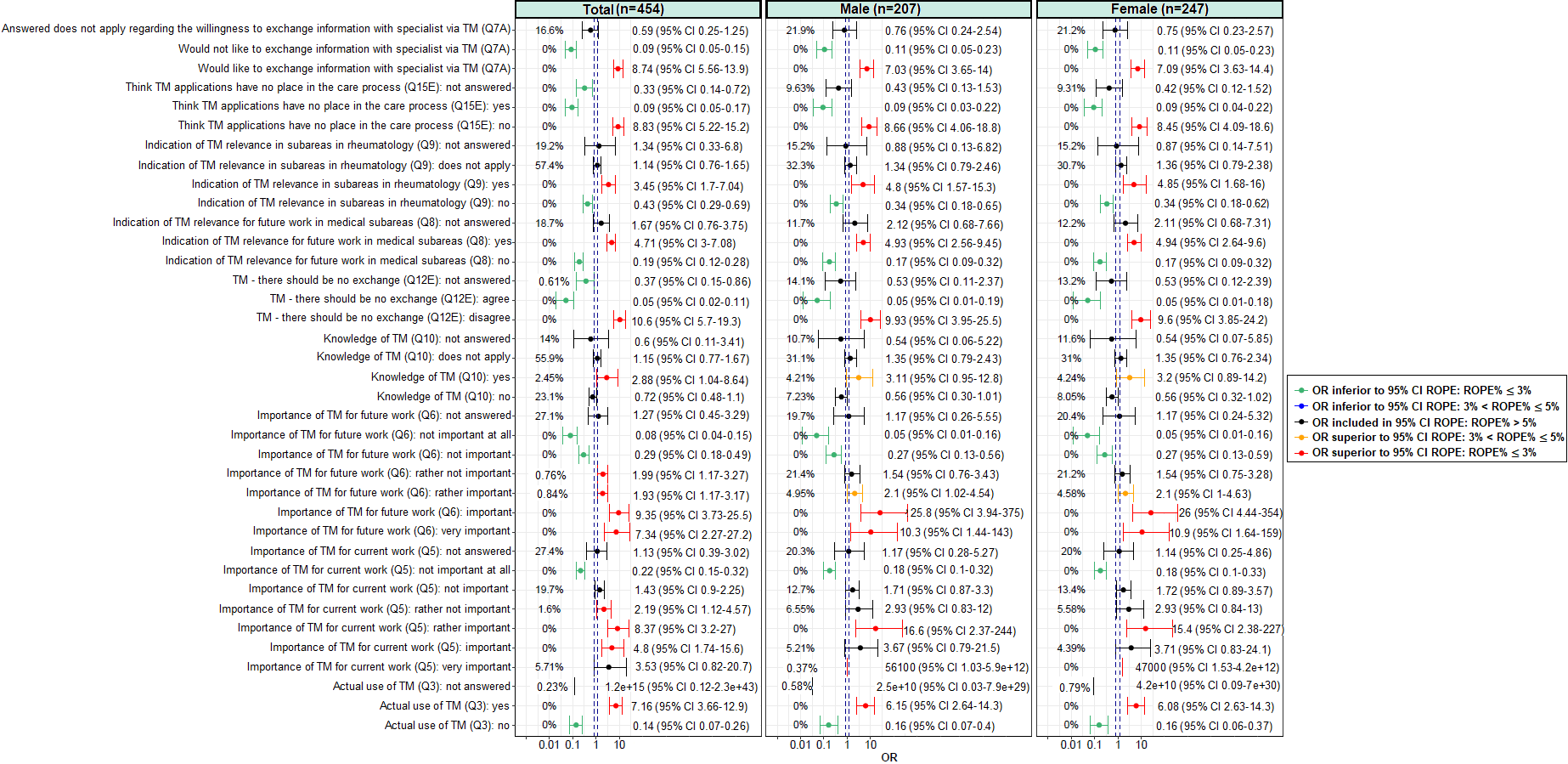


Figure S6. Bayesian univariate logistic regression: relationship between the willingness to use telemedicine and work characteristics—part 2.


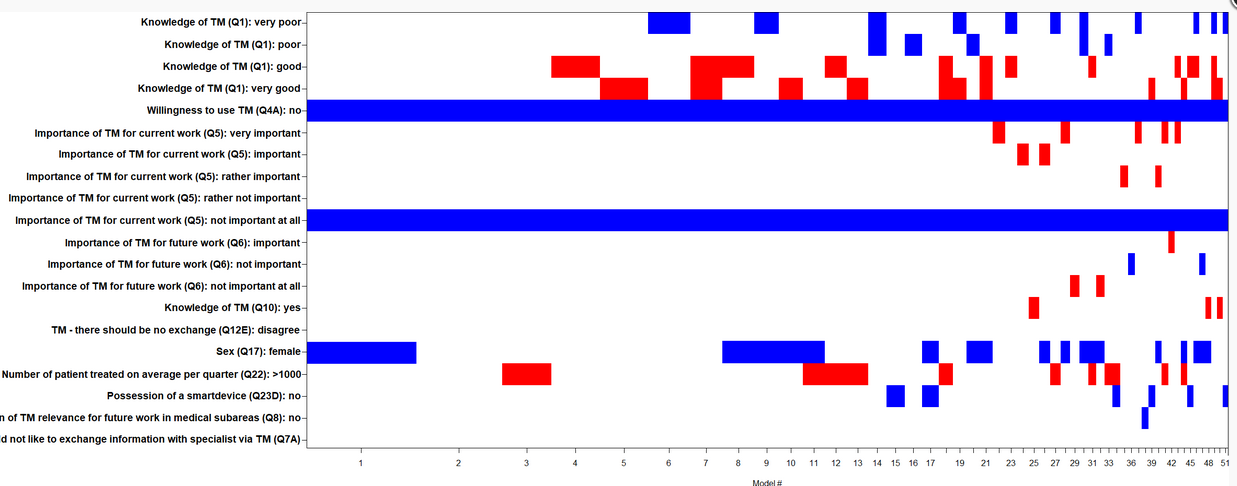


Figure S7. Bayesian model averaging analysis: results for the actual use of telemedicine in both sexes.


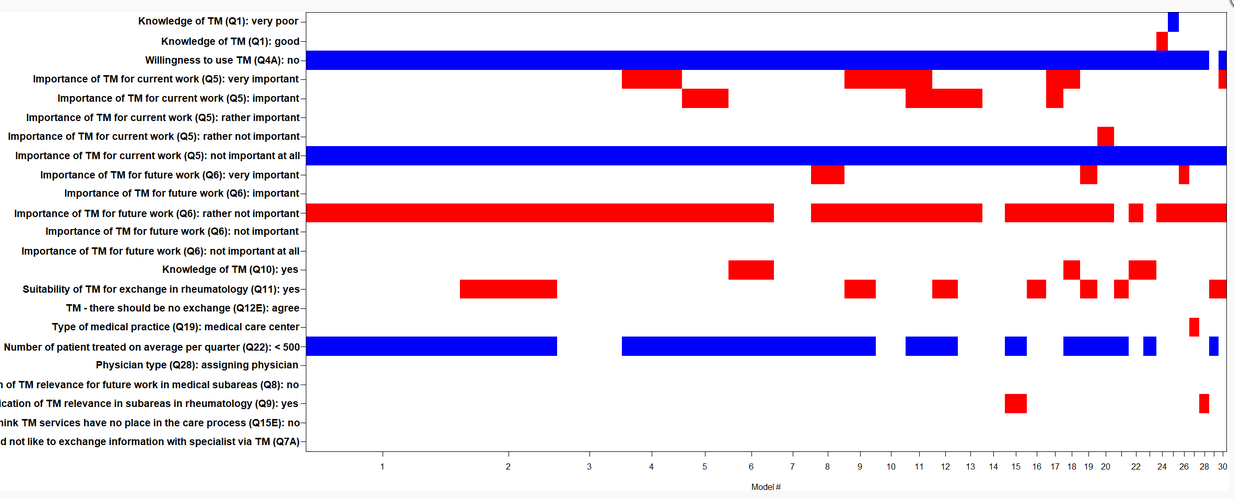


Figure S8. Bayesian model averaging analysis: results for the actual use of telemedicine in male physicians.


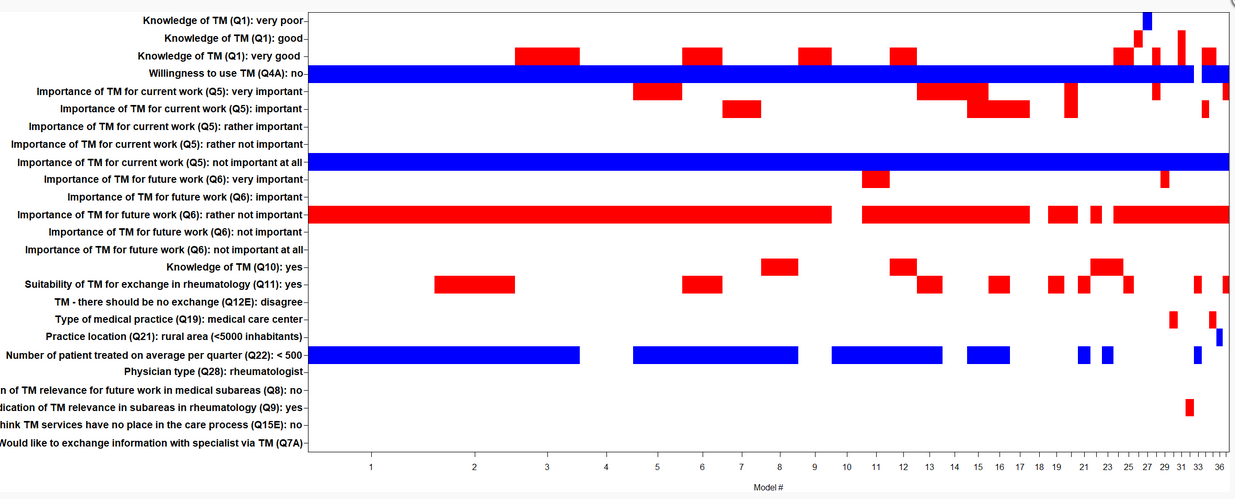


Figure S9. Bayesian model averaging analysis: results for the actual use of telemedicine in female physicians.


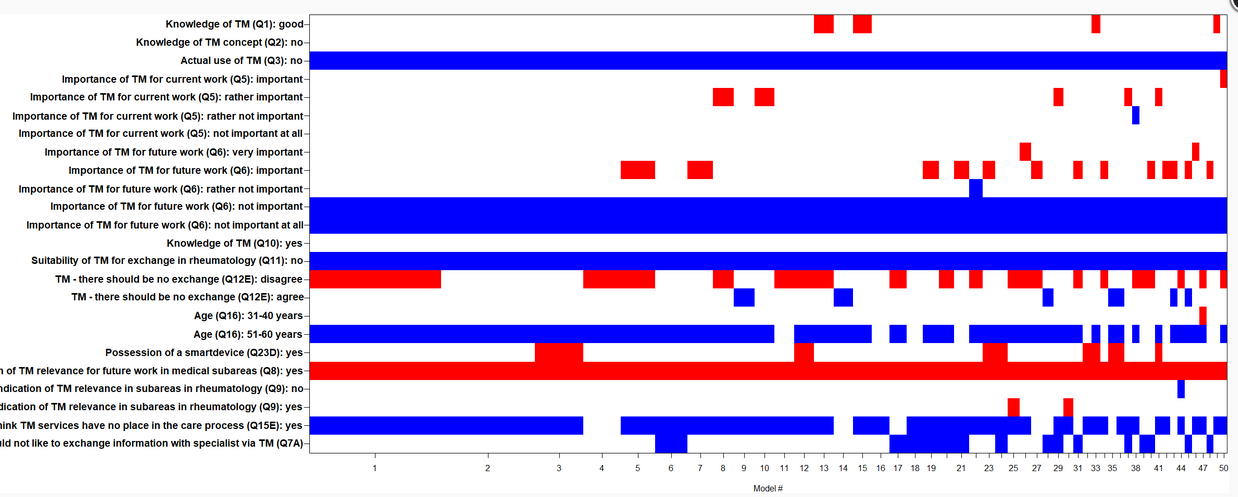


Figure S10. Bayesian model averaging analysis: results for the willingness to use telemedicine in both sexes.


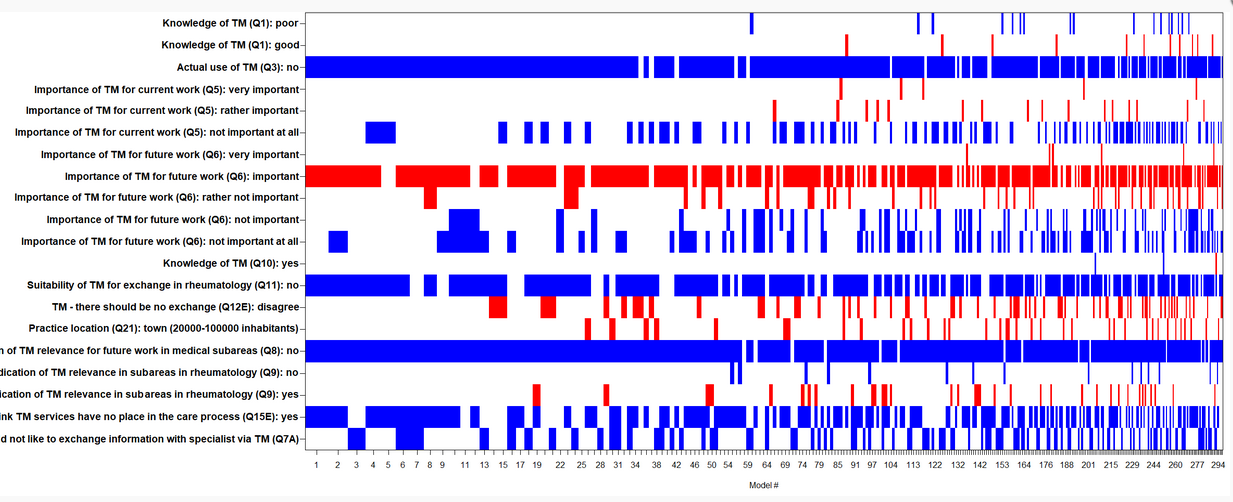


Figure S11. Bayesian model averaging analysis: results for the willingness to use telemedicine in male physicians.


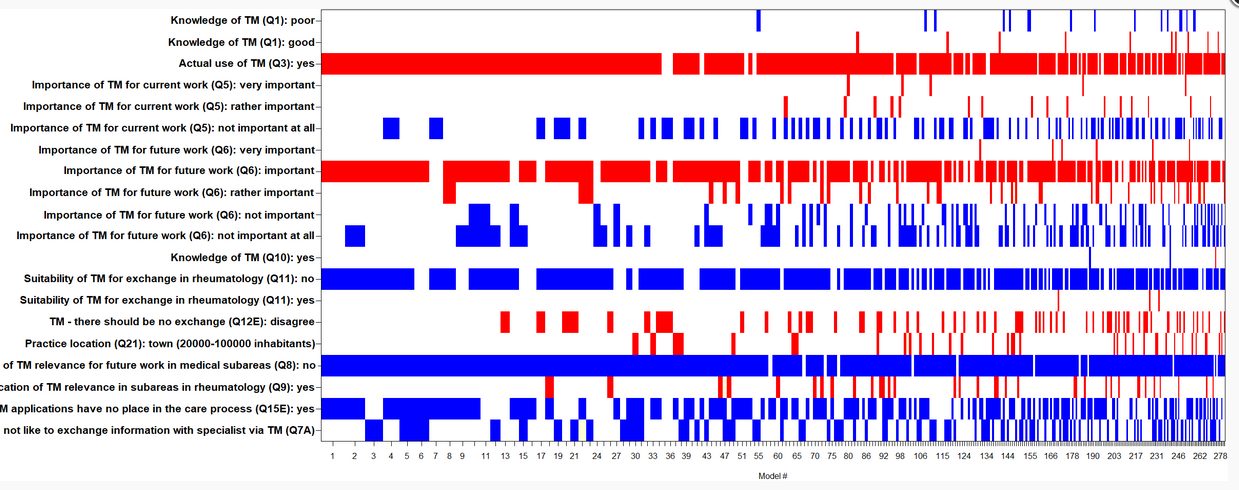


Figure S12. Bayesian model averaging analysis: results for the willingness to use telemedicine in female physicians.
